# Editorial: World digestive health day: investigating the link between neurodegenerative disease and gut microbiota

**DOI:** 10.3389/fnagi.2023.1351855

**Published:** 2024-01-03

**Authors:** Shampa Ghosh, Sunil Dhungel, Mohd. Farooq Shaikh, Jitendra Kumar Sinha

**Affiliations:** ^1^GloNeuro, Noida, Uttar Pradesh, India; ^2^ICMR-National Institute of Nutrition (NIN), Tarnaka, Hyderabad, India; ^3^Department of Neurology and Neuroscience, Medical University of Americas, Charlestown, Nevis, Saint Kitts and Nevis; ^4^Neuroscience Society of Nepal, Gwarko, Lalitpur, Nepal; ^5^School of Dentistry and Medical Sciences, Charles Sturt University, Orange, NSW, Australia

**Keywords:** neurodegenerative diseases, gut-brain axis, microbiome, Alzheimer's disease, Parkinson's disease, neuroinflammation, blood-brain barrier, short-chain fatty acids

Neurodegenerative diseases (NDs), such as Alzheimer's and Parkinson's disease (PD), are debilitating conditions that affect millions of people worldwide (Ghosh et al., 2020). Research has shown that there is a potential link between these diseases and the gut microbiota, which is the collection of microorganisms that live in our digestive tracts. This link is known as the gut-brain axis, which suggests that the gut and the brain are interconnected and can influence each other's function. In recent years, there has been growing interest in investigating the mechanisms that underlie this relationship and the potential implications for treating and preventing NDs. Through this Research Topic, we tried to examine the current state of research on the link between neurodegenerative disease and gut microbiota. Specifically, we explored the gut-brain axis and its relevance to these diseases, the potential mechanisms that link gut microbiota and NDs, and the key findings from recent studies investigating this link. We hope future research will provide a better comprehension of the liaison of NDs and gut microbiota and the potential implications for future research and treatment.

## The relationship between neurodegenerative disease and gut microbiota

The gut-brain axis is a two-way communication system between gut microbes and the brain that coordinates the activities of the gastrointestinal tract and the central nervous system (CNS) (Kaviyarasan et al.). It involves cross-talk between the gut and the brain and is involved in bidirectional communication between the two systems. This axis is related to NDs such as Parkinson's and Alzheimer's (Kaviyarasan et al.). The microbiome can affect brain-derived neurotrophic factor (BDNF), N-methyl-D-aspartate receptor (NMDAR), and neuroinflammation through direct neural, neuroendocrine, and immunological mechanisms (Singh et al., 2022). In fact, lower levels of BDNF were found in AD brains, and mice lacking BDNF exhibit altered gastrointestinal tract innervation development (Singh et al., 2022). Furthermore, exposure to antibiotics is one of the most potent reasons for disturbing gut microbiota, which is crucial to maintaining the gut-brain axis's proper functioning (Sheng et al.). The composition and metabolites of the microbiota have potential as novel therapeutic interventions for chronic diseases, including NDs (Sheng et al.). However, more studies are required to better understand the underlying mechanisms of the gut-brain axis and its relation to NDs, from bench to clinical research.

## Potential mechanisms linking gut microbiota and neurodegenerative diseases

The gut microbiota's dysregulation and subsequent neuroinflammation may play a crucial role in the pathogenesis of neurodegenerative diseases such as AD (Lee et al.). Observational studies have shown that AD patients have a decreased gut microbiome diversity, which could contribute to the development of AD (Lee et al.). The gut microbiota modulates gut permeability and various immune functions by secreting toxins and short-chain fatty acids, which ultimately affect brain health (Lee et al.). The gut-brain axis is a pathway of communication between the gut microbiota and the brain, and can occur through endocrine, immune, and nervous systems (Ghosh et al., 2019). Alterations in the gut microbiota composition can influence brain disorders, such as AD, via the microbiota-gut-brain axis. Certain bacteria in the human gut, including *Staphylococcus aureus, Escherichia coli, Salmonella, Mycobacterium, Klebsiella pneumoniae*, and *Streptococcus*, promote the production and aggregation of amyloid-β (Aβ) protein in the enteric nervous system (Wu et al., 2021). The bacterial amyloid protein produced by coccus-shaped bacteria can activate the innate immune system, triggering responses by Toll-like receptors and cluster of differentiation 14, resulting in inadequate recognition of misfolded Aβ and decreased Aβ clearance, followed by the production of cytokines leading to intestinal disturbances (Wang et al., 2022). Dysregulation of the gut microbiota leads to oxidative stress, inflammation, disruption of the blood-brain barrier (BBB), activation of the immune system, neurofibrillary tangles, and Aβ plaques followed by neurodegeneration (Wang et al., 2022). The BBB's integrity is harmed by the secretion of biomolecules from gut microbiota, which worsens with increased dysbiosis. The gut microbiota contains beneficial bacteria that help maintain redox homeostasis, while dysregulation of the gut microbiota has been strongly associated with the development of oxidative stress-mediated NDs (Wang et al., 2022). These findings suggest that modulation of AD through dietary and gut microbiota interventions may be potential therapeutic strategies.

## Research on the link between gut microbiota and neurodegenerative diseases

Recent studies have shown that there may be a link between gut microbiota and neurodegenerative diseases such as AD and PD (Gubert et al., 2022). Alterations in gut microbiota may contribute to the progression of these diseases, as the gut microbiota plays a role in modulating inflammation in NDs. Targeting the gut microbiota could be a potential therapeutic strategy for the treatment of neurodegenerative disorders, and there is potential for different microbiota-based therapeutic strategies to prevent, modify, or halt the progress of neurodegeneration (Gubert et al., 2022). However, a causal role for the gut microbiota in neurodegeneration is yet to be confirmed. The gut microbiome may act as an intermediate factor between the host and the environment affecting key aspects in the neurodegeneration process such as inflammation and protein homeostasis (Kaviyarasan et al.). Research has shown that gut dysbiosis mechanisms can lead to neuroinflammation and neurodegeneration in AD, PD, amyotrophic lateral sclerosis, and Huntington's disease (Khatoon et al.). Gut microbiota influences the metabolism of neuroactive compounds implicated in NDs, including short-chain fatty acids (SCFAs) (Gubert et al., 2022). Studies have also shown that fecal SCFA concentrations in individuals with PD are lower compared to healthy controls (Sheng et al.). These findings indicate that the role of gut microbiota in the development and progression of neurodegenerative diseases may be significant. Further research is needed to better understand the mechanisms underlying this relationship, but given the potential applications for new therapeutics, it is a promising area of study for future research.

The findings and discussions in this Research Topic provide evidence of a link between gut microbiota and NDs. This axis influences the metabolism of neuroactive compounds implicated in NDs and may play a significant role in their development and progression. The integrity of the blood-brain barrier is also affected by the secretion of biomolecules from gut microbiota, which worsens with increased dysbiosis. Modulation of NDs and other brain disorders through dietary and gut microbiota interventions may be potential therapeutic strategies (Ghosh et al., 2023). However, further research is needed to better understand the mechanisms underlying this relationship and to identify effective interventions. Limitations of this study include the need for larger sample sizes and more diverse populations. Future research should focus on identifying the specific microbiota species and metabolites involved in the gut-brain axis and their effects on neurodegenerative diseases. Overall, this promising area of study has the potential to lead to the development of new therapeutics for neurodegenerative diseases.

## Author contributions

SG: Conceptualization, Investigation, Methodology, Visualization, Writing—original draft, Writing—review & editing. SD: Writing—review & editing. MS: Writing—review & editing. JS: Conceptualization, Funding acquisition, Investigation, Resources, Supervision, Visualization, Writing—original draft, Writing—review & editing.
